# Model-Informed Repurposing of Medicines for SARS-CoV-2: Extrapolation of Antiviral Activity and Dose Rationale for Paediatric Patients

**DOI:** 10.3390/pharmaceutics13081299

**Published:** 2021-08-19

**Authors:** Federico Romano, Salvatore D’Agate, Oscar Della Pasqua

**Affiliations:** 1 Clinical Pharmacology & Therapeutics Group, University College London, London WC1H 9JP, UK; federico.romano.17@ucl.ac.uk (F.R.);; 2 Clinical Pharmacology Modelling and Simulation, GlaxoSmithKline, Brentford TW8 9GS, UK; 3 Bioinformatics and Computational Biology, Istituto per le Applicazioni del Calcolo, Consiglio Nazionale delle Ricerche, 00185 Rome, Italy

**Keywords:** dose rationale, paediatrics, COVID-19, SARS-CoV-2, repurposing, model-informed drug development, MIDD, antiviral drugs, remdesivir

## Abstract

Repurposing of remdesivir and other drugs with potential antiviral activity has been the basis of numerous clinical trials aimed at SARS-CoV-2 infection in adults. However, expeditiously designed trials without careful consideration of dose rationale have often resulted in treatment failure and toxicity in the target patient population, which includes not only adults but also children. Here we show how paediatric regimens can be identified using pharmacokinetic-pharmacodynamic (PKPD) principles to establish the target exposure and evaluate the implications of dose selection for early and late intervention. Using in vitro data describing the antiviral activity and published pharmacokinetic data for the agents of interest, we apply a model-based approach to assess the exposure range required for adequate viral clearance and eradication. Pharmacokinetic parameter estimates were subsequently used with clinical trial simulations to characterise the probability target attainment (PTA) associated with enhanced antiviral activity in the lungs. Our analysis shows that neither remdesivir, nor anti-malarial drugs can achieve the desirable target exposure range based on a mg/kg dosing regimen, due to a limited safety margin and high concentrations needed to ensure the required PTA. To date, there has been limited focus on suitable interventions for children affected by COVID-19. Most clinical trials have defined doses selection criteria empirically, without thorough evaluation of the PTA. The current results illustrate how model-based approaches can be used for the integration of clinical and nonclinical data, providing a robust framework for assessing the probability of pharmacological success and consequently the dose rationale for antiviral drugs for the treatment of SARS-CoV-2 infection in children.

## 1. Introduction

The global spread of the SARS-CoV-2 virus has impelled clinical drug developers, and academic investigators worldwide to explore the efficacy of novel candidates and existing medicinal products with antiviral and anti-inflammatory properties for the treatment of coronavirus disease 2019 (COVID-19). The race for the approval and implementation of clinical trials prompted by the urgent medical need has allowed for empirical choices of the drug, dose, and dosing regimens to be tested in these trials. Without an established standard of care, and detailed understanding of the factors determining disease progression, different groups of patients have been enrolled into clinical protocols, aimed at characterisation of treatment efficacy and safety at different stages of the disease, from the onset of the infection to acute respiratory distress syndrome. It is worth noting that in this process very limited attention was given to the role of quantitative clinical pharmacology principles as a tool for translation, integration, and extrapolation of pharmacokinetic and pharmacodynamic data [1,2]. Such a scientific rift has resulted in randomised controlled and observational clinical trials of questionable scientific quality, including interventions that had poor dose rationale.

However, these efforts have focused primarily on the evaluation of efficacy in adult and elderly patients, under the assumption that the paediatric population was less vulnerable to the virus. In fact, it was initially reported that children with COVID-19-related severe acute respiratory syndrome developed less severe disease and had far lower mortality rates [3,4,5]. Variant strains of SARS-CoV-2 have since appeared, having higher infectivity rates and posing a greater risk to children. This increases the chances of progression and clinical presentation, from mild symptoms to severe inflammatory syndrome [6,7], particularly in children with underlying health conditions. Among these, it is worth mentioning immunodeficiencies, agammaglobulinemia, Bruton’s disease, Wiskott-Aldrich syndrome, cystic fibrosis as well as those undergoing therapy for haemato-oncology. Another vulnerable group includes preterm new-borns with associated pulmonary fibrosis or dysplasia [8]. Hence, regardless the development and approval of vaccines and monoclonal antibodies in some countries, there is a critical need for cost-effective antiviral therapies, which remain limited with the evolving pandemic [9].

To date, no new SARS-CoV-2-specific antivirals have been identified for adult or paediatric use. Similarly, there is a lack of good-quality data from clinical trials for repurposed antiviral drugs in children, with the management in children remaining essentially supportive, despite the increased prevalence of variants of concern in adolescents and young adults. Of the drugs that have been tested, only remdesivir (RDV) has been approved for treating severe infection in children, above the age of 12 [10]. Given that paediatric groups are not being prioritised for vaccinations by governments, there is a need to ensure a robust dose rationale and appropriate characterisation of the efficacy and safety profile of drugs that could be used in children, especially in patients younger than 12 years of age, for whom the benefit–risk balance of vaccination remains uncertain.

For promising antiviral candidates, repurposing should be based on an understanding of the dose–exposure–effect relationships prior to any testing in a clinical setting. However, despite the relevance of pharmacokinetic–pharmacodynamic (PKPD) principles for establishing the efficacy and safety profile of a repurposed product, the selection and suitability of dose regimens of the vast majority of investigational medicinal products tested in clinical trials has been empirical [11]. Unfortunately, the COVID-19 pandemic has not been an exception. As of 16 September 2020, there were 2553 COVID-19/SARS-CoV-2 clinical trials recorded in clinicaltrials.gov, of which 14% were Phase III studies in adult subjects. Of the remaining trials, only 28% were dose ranging Phase II studies. Most of these studies have been terminated for safety or futility reasons, as they failed to demonstrate efficacy.

Clearly, the rationale for a dose and dosing regimen is not a matter of trial and error, nor should it be based on tolerability (e.g., the maximum tolerated dose). Here we aim to demonstrate how a model-based approach can be used as a tool for extrapolating information across populations, and consequently support the dose rationale and associated go/no-go decisions. In addition to maximising data integration, the use of quantitative clinical pharmacology methods enables the prediction of pharmacokinetics, pharmacodynamics, and the probability of pharmacological success in the target patient population. More specifically, three candidate molecules (RDV, chloroquine (CQ), and hydroxychloroquine (HCQ)), which have been previously tested for the treatment of adult patients with mild or moderate COVID-19 symptoms are used as examples of the approach for the evaluation of potential antiviral treatments for children.

It is our endeavour to show that irrespective of the evidence for substantial antiviral activity and a favourable benefit–risk profile in another indication, the dose selection for the paediatric population cannot be based on empirical extrapolation of findings without careful consideration of pharmacokinetics and other relevant factors that may determine differences in the treatment response, such as immunocompetence. Moreover, given the vulnerability of the target population, dose ranging studies should be avoided in children if there is evidence of the underlying PKPD relationship for the antiviral activity. Instead, different doses and dosing regimens could be tested in silico using clinical trial simulations to predict the therapeutically relevant dose range of the drug of interest based on target attainment concepts, which provide insight into how changes in drug exposure in specific organs and tissues may affect the overall efficacy and safety profile of the treatment. In addition, covariates can also be evaluated to assess the impact of influential factors on pharmacokinetics or pharmacodynamics [10]. The same concepts can be applied for novel candidate molecules, for which clinical data are not available.

Our objectives were three-fold (1) to illustrate how population pharmacokinetic modelling and extrapolation can be used in conjunction with clinical trial simulations to describe the systemic and putative target tissue exposure in a paediatric population receiving three drugs previously proposed for the treatment of adult COVID-19 patients with mild and moderate symptoms, namely CQ and HCQ and RDV; (2) to assess the probability target attainment (PTA) following different doses and dosing regimens; and (3) to compare simulated plasma levels to reported safety thresholds to support go/no-go decision to proceed with the implementation of a clinical trial.

## 2. Materials and Methods

### 2.1. General Modelling and Simulation Methodology

**Figure 1:**
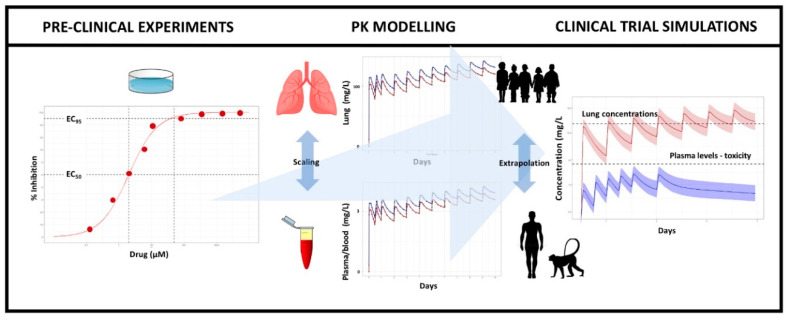
Framework for the evaluation of the antiviral activity of repurposed drugs for the treatment of SARS-CoV-2 infection in paediatric patients. **Pre-clinical experiments**—Antiviral activity estimated in vitro is used as reference for the target exposure range to be achieved in vivo in pre-clinical species and in humans. **Pharmacokinetic modelling**—Compartmental pharmacokinetic models are used to characterise or predict the pharmacokinetics in the relevant matrix (e.g., plasma, blood, cells) in pre-clinical species and in humans. Scaling factors can be used in conjunction with distributional models, taking into account protein binding if required, to estimate tissue or target organ exposure. In the context of SARS-CoV-2, the lung was considered as the primary organ of interest. Allometric principles or physiologically-based pharmacokinetic modelling are subsequently applied to extrapolate pharmacokinetic properties from pre-clinical species or adult subjects to children. Here we relied on allometric principles under the assumption that body weight is the main covariate factor affecting drug disposition. **Clinical trial simulations (CTS)**—The availability of a virtual cohort of patients provides the basis for the evaluation of different doses and dosing regimens in the target patient population. Using appropriate sampling and resampling techniques, it is possible to obtain virtual patients with baseline characteristics that reflect real subjects. CTS is performed using the selected virtual patient population in conjunction with the pharmacokinetic model parameters obtained from the extrapolation step. A range of scenarios can then be considered to assess treatment performance. Scenarios included simulation of the concentration vs. time profiles of each drug over a period of up to 14 days, taking into account the dosing regimens currently recommended for approved indications in adults and known time course of the SARS-CoV-2 infection in humans. Decision criteria are applied to the results to ensure systematic evaluation of the data. Here, the main go/no-go decision criterion to proceed to the implementation of a clinical trial was evidence of exposure levels that are associated with at least 95% in-vitro viral inhibition. A second criterion was the anticipated (drug-related) adverse event profile associated with systemic drug exposure, if known. For drugs with limited safety data, the maximal tested dose was set to that which has been approved by regulatory agencies.

An outline of the various steps required for the implementation of a model-based approach for evaluation of the dose rationale for repurposed drugs is presented in Figure 1. All modelling and simulation steps were performed using nonlinear mixed effects modelling, as implemented in NONMEM v. 7.3 (ICON Development Solutions LLC, Ellicott City, MD, USA) using PsN v.4.8.1 (Uppsala University, Uppsala, Sweden). In order to develop a pharmacokinetic model for RDV, the first-order conditional estimation method with interaction (FOCE-I) was used for model building. Graphical evaluation was undertaken using R v.3.5.3 (R Core Team, Vienna, Austria). Parameters were retained if the forward addition led to a decrease in the objective function value (OFV) > 3.84 units (*p* < 0.05). The final model selection was based on biological plausibility, OFV criteria, residual standard error (RSE) of the parameter estimates, whether convergence occurred and the adequacy of goodness-of-fit (GOF) plots.

### 2.2. Pre-Clinical Data, Modelling, and Extrapolation Assumptions

All in vitro experimental data retrieved for the purpose of this analysis were conducted using African green monkey renal epithelial Vero cells. A concise description of the experimental protocols used to generate these data can be found in [12,13,14,15,16]. Data from human airway epithelial cells were also used for the evaluation of the antiviral activity of RDV. Details on the mechanisms of action associated with the antiviral activity of CQ [17,18,19,20,21], HCQ [22], and RDV [23,24,25] along with the reference target concentrations selected from the available publications are summarised in Table 1. Even though genotypic and/or phenotypic differences between human lung tissue and experimental protocol conditions cannot excluded, it was assumed that SARS-CoV-2 growth in Vero cells largely reflect viral dynamics in humans. In addition, as exposure–response curves describing antiviral activity in vivo were not available, it was hypothesized that in vitro PKPD relationships accurately describes the viral dynamics and drug effects in vivo.

Whilst CQ and HCQ have been directly associated with antiviral activity against SARS-CoV-2, it is the metabolite of RDV that shows antiviral activity. RDV was therefore further assumed to be primarily metabolised into the main nucleoside metabolite (GS-441524), with fast equilibration between plasma and intracellular (tissue) levels, where it is converted into the pharmacologically active triphosphate form (GS-443902). GS-443902 has been shown to inhibit viral RNA polymerases [24], with a broad spectrum of activity against coronaviruses. In the absence of clinical data, our analysis was performed assuming similar metabolic ratio in macaques and humans [26]. This assumption was based on data showing comparable expression of plasma hydrolases across species [27].

From a pharmacokinetic perspective, our working hypothesis was that tissue distribution and equilibration kinetics were achieved rapidly, despite disease progression in some patients with important inflammatory activity. Hence, tissue partition coefficients were used to derive the blood or plasma to lung ratios and assess drug concentrations in tissue [23]. In addition, where available, lung tissue concentrations were extrapolated from pre-clinical species to humans. As there is no evidence of active transport mechanisms, unbound or free fraction in all compartments (plasma, extracellular, and intracellular) were considered to be identical [28].

Another important pharmacokinetic consideration for the extrapolation of in vitro findings was metabolic stability. Both CQ and HCQ are metabolised via cytochrome P450 enzymes in the liver. However, CQ has also been shown to be metabolised in vitro to its main metabolite in a predictable manner, with HCQ assumed to show a similar pattern [29,30]. Whilst both drugs have pharmacologically active metabolites as anti-malarials [31], without data to support this for SARS-CoV-2, the overall antiviral activity of CQ and HCQ was based on the parent compounds. In contrast, RDV and its parent nucleoside analogue GS-441524 exhibit anti-viral activity against coronaviruses [13,32] by incorporating into the replicating RNA chains. This causes premature chain termination during transcription, thus interrupting subsequent replication [33,34].

Differently from CQ and HCQ, pharmacokinetic data in humans were not available for RDV at the time of this investigation. As the RDV concentration vs. time profiles indicate very fast elimination kinetics in non-human primates due to hydrolysis and subsequent formation of the main metabolite GS-441524 [35,36], we used GS-441524 to extrapolate the pharmacokinetics of RDV to humans. Importantly, similar plasma exposures for RDV and GS-441524 have been shown between healthy rhesus monkeys and human subjects [37,38]. In addition, in our work, we have assumed fast equilibration between plasma and intracellular (tissue) levels of the active metabolite [39]. This assumption allowed us to treat concentration data for GS-441524 as a surrogate for intracellular levels of the pharmacologically active triphosphate metabolite GS-443902.

### 2.3. Pharmacokinetic Modelling and Paediatric Extrapolation

The pharmacokinetics of CQ in blood and HCQ in blood and plasma was evaluated using pharmacokinetic models previously published based on adult subjects [40,41,42,43]. To allow the characterisation of lung and plasma concentrations, a ratio of 5 was assumed for the correlation between whole blood-and-plasma levels of CQ [44]. For RDV, a pharmacokinetic model was developed based on the mean plasma concentration-time profile of the main nucleoside metabolite (GS-441524) in healthy rhesus macaques following intravenous administration of 10 mg/kg of RDV. Full details of the experimental protocol can be found in [23]. A diagram of the model structure and parameter estimates for CQ, HCQ, and RDV used for the characterisation of the systemic and lung exposure in children is presented in Supplementary Materials, (see Figures S1–S4 and Tables S1–S4). Given the use of GS-441524 as basis for the characterisation of the relevant target exposure, and the assumption of complete conversion of RDV into GS-441524 prior to the formation of the triphosphate metabolite (GS-443902), a sensitivity analysis was performed in which GS-441524 formation and elimination rates were up to 2-fold larger or smaller than those used in the initial estimation procedures.

As the objective of the sensitivity analysis was to assess the potential implications of unknown precision of the parameters derived from mean concentration vs. time profiles, the range of variation was limited to the observed data distribution (based on mean ± standard deviation). Results from this evaluation provided further confidence of the results obtained subsequently in the simulation scenarios, which also included additional random interindividual variability in disposition parameters (i.e., clearance and volume of distribution) of 30%.

For all models, pharmacokinetic disposition in the paediatric population was scaled using fixed allometric exponents on clearance and volume of distribution, normalised to a 70 kg adult. Predictive performance of the model was assessed by comparing predicted exposure and secondary pharmacokinetic parameters with previously published data, if available.

### 2.4. Prediction of Drug Exposure at the Target Organ

Different assumptions and data sources were used to predict lung tissue concentrations. Average blood-to-lung tissue ratio was used to predict intracellular concentrations in lung tissue for CQ (ratio = 1:50) and HCQ (ratio = 1:29) By contrast, for RDV plasma concentrations of the metabolite GS-441524 were used for the prediction of lung exposure. This was based on the findings that GS-441524 concentrations in murine plasma and lung were similar, and did not accumulate in the lung [45]. As our model was developed using micromolar concentrations, concentrations were subsequently converted into mg/L using the molecular weight of GS-441524. The aforementioned data were deemed suitable as basis for inference about lung distribution under the assumption that age and organ size should not alter equilibration kinetics. In addition to disease-related changes in perfusion and vascular permeability [46], plasma protein binding was not considered relevant for tissue distribution, on account of the low plasma protein binding for CQ, HCQ, and RDV. These ratios were used with the simulated profiles in plasma or blood to generate the concentration vs. time profiles of the moieties in the lung.

### 2.5. Clinical Trial Simulations (CTS)

The primary goal of the CTS was to establish the dose rationale for CQ, HCQ and RDV in the paediatric population. Data sets for simulations were created using baseline demographic characteristics from 963 subjects (age range: 2–17 years, median age 10 [IQR 6–13] years) retrieved from the US National Centre for Health Statistics (NHANES) databases [47]

The age range for the paediatric population used here (i.e., from 2 to <18 years old) was based on epidemiological data on the prevalence and severity of SARS-CoV-2 infection [3], taking into account the currently approved indications of the drugs under investigation. Whilst CQ and HCQ are approved for paediatric use as anti-malarials, RDV has not been previously tested in children. Based on the summary of product characteristics, CQ can be used in children from 1 year of age, whilst the use of HCQ is limited to older subjects (>30 kg body weight) due the lack child-friendly formulations.

Based on the known metabolic pathways of CQ, HCQ, and RDV, it was assumed that ontogeny and other maturational processes would have minor effect on drug disposition. Consequently, age-related differences were driven by interindividual variation in body weight (range: 12.5–100 kg, median 39.4 [IQR 23.3–58.25] kg), which was parameterised according to allometric principles with fixed exponents on clearance(s) and volume(s) of distribution. The validity of this assumption was partly assessed by the predictive performance of the models. We have attempted to establish the predictive performance by comparing model-predicted exposure to observed data in paediatric patients. A few references were identified for such a comparison [48,49,50].

Concentration vs. time profiles were simulated following administration of the doses and dosing regimens summarised in Table 2. As sample size was considered sufficiently large to allow characterisation of the effect of covariates and interindividual variability on clearance and volume of distribution, simulation replicates were performed only to confirm the precision of the simulated concentration vs. time profiles. Body weight and age distributions in the simulation data sets reflect the data distribution in the NHANES database.

Different factors were considered for the dose rationale for the paediatric population. Dosing regimens for currently approved indications (i.e., CQ, HCQ) and ongoing development (RDV) were selected as reference with regard to the overall safety profile. (see Table 2). Simulated scenarios included only weight-based dosing regimens, which was assumed to correct for the differences in disposition properties due to changes in body size. Lastly, we assumed that the range of in vitro potencies previously reported represented the entire variability that is expected from experimental cell-based assays [51].

Simulated concentration vs. time profiles were obtained according to a rich sampling scheme for each subject, including a sampling time every hour for the entire duration of the treatment. Secondary pharmacokinetic parameters (area under the concentration vs. time curve [*AUC*], maximum concentration [*Cmax*] and trough concentration [*Cmin*]) were subsequently calculated and summarised using descriptive statistics. *AUC* was calculated using the trapezoidal method, whereas *Cmax* and *Cmin* were obtained directly from the predicted concentrations.

### 2.6. Go/No-Go Criteria

The probability of target attainment (PTA) along with safety thresholds were defined as go/no-go decision criteria for subsequent evaluation of the selected dosing regimens in a prospective clinical trial. To take into account the interindividual variability, precision and eventual bias in the reported pre-clinical PKPD parameter estimates when evaluating the different doses and dosing regimens, simulations were implemented including best and worst case scenarios, whenever possible. These scenarios were based on the lowest and highest reported values for the antiviral activity (expressed as IC_50_, i.e., in vitro potency). For CQ and HCQ, the PTA was based on the predicted lung exposure at 24-, 72- and 168-h post-dose. On the other hand, for RDV, the PTA was based on predicted average steady state concentrations in plasma under the assumption of fast equilibration kinetics between plasma and lung tissue.

### 2.7. Safety Thresholds

Given the risk of toxicity associated with high doses of the compounds under evaluation, safety thresholds were also included as go/no-go criteria. Exposure levels corresponding to known adverse events were used for CQ [52]. In contrast, the safety of HCQ was found to be less well characterised in the literature. Serious toxicity has been reported previously in patients with plasma levels between 0.64 to 6.1 mg/L [53], but the therapeutic range for the treatment of systemic lupus erythematosus overlaps significantly with those values (0.5–2 mg/L) [54]. Therefore, simulation scenarios were implemented including best and worst case, with a safety threshold of 0.64 mg/L as the worst-case and 2 mg/L as the best-case scenario for HCQ. Because of the limited clinical experience with RDV, the maximum allowed exposure was set to the dose and dosing regimen previously evaluated for the Ebola virus (see Table 2) [55].

## 3. Results

### 3.1. Predicted Pharmacokinetic Profiles in Paediatric Patients

Simulated profiles were obtained for each drug using a population pharmacokinetic model, including allometric principles with fixed allometric exponents for disposition parameters. An overview of the predictive performance of the models for CQ and HCQ is presented in Tables S5 and S6, respectively (see Supplementary Materials). Predicted pharmacokinetic profiles and systemic concentrations for CQ and HCQ were in agreement with previously published data following administration of both drugs to paediatric patients at a dose range comparable to that included in the simulation scenarios.

For RDV, a two-compartment model including the effect of body weight on drug disposition was found to best characterise the pharmacokinetic data. Final parameter estimates are reported in the Supplementary Materials. A comparison between model-predicted and observed secondary pharmacokinetic parameters [38] is shown in Table S7. A visual predictive check comparing model predictions and previously reported data [56] is presented in Figure S5. These results are further complemented by model-predicted secondary pharmacokinetic parameters for GS-441524 in children (Tables S8 and S9). Given the assumption that clearance and metabolite formation rates vary according to allometric principles, one cannot exclude the possibility that bioavailability or metabolite formation clearance may differ across species. As precision and uncertainty in parameter estimates could not be obtained from the extrapolation based on a mean pharmacokinetic profile, a sensitivity analysis was performed to explore how over- or under-prediction of pharmacokinetic properties affects the conclusions of the analysis. The pharmacokinetic profiles of GS-441524 over time assuming lower and higher elimination rate constants in adults is depicted in Figure S6. Results show that even a 2-fold lower elimination rate of GS-441524 2-does not warrant a high probability of target attainment in the paediatric population (Table S10).

### 3.2. Simulation Scenarios for Selected Doses and Dosing Regimens

**Figure 2:**
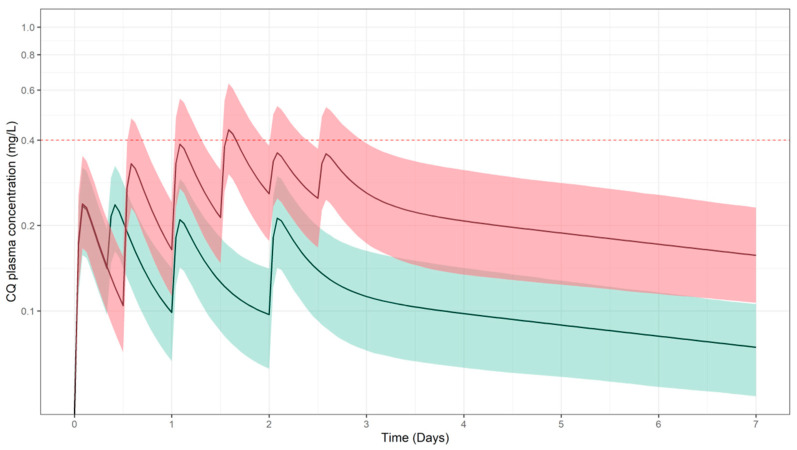
Simulated plasma CQ concentration vs. time profiles following oral administration of total CQ doses of 25 mg/kg (green) and 50 mg/kg (red). Solid lines and shaded areas depict the predicted median profile and corresponding 95% confidence intervals, respectively. Dotted horizontal line represents the reported lower bound safety threshold in plasma of 0.4 mg/L, as reported in [52]. Results refer to a paediatric population (*n* = 963), with a body weight range between 12.5 and 100 kg. Dose and schedule details are summarised in Table 2.

**Figure 3:**
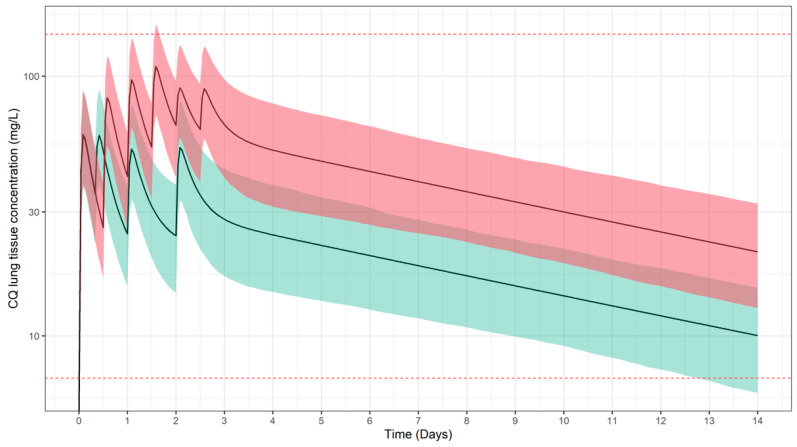
Simulated CQ concentration vs. time profiles in lung tissue following oral administration of total CQ doses of 25 mg/kg (green) and 50 mg/kg (red). Solid lines and shaded areas depict the predicted median profile and corresponding 95% confidence intervals, respectively. Dotted horizontal lines represent the lowest (4.59 mg/L) and highest (110.5 mg/L) estimates for IC_95_ derived from [12,14]. Results refer to a paediatric population (*n* = 963), with a body weight range between 12.5 and 100 kg. Dose and schedule details are summarised in Table 2.

In Figure 2, CQ plasma concentration vs. time profiles were simulated for regimens that included a high dose (50 mg/kg, red shaded area), as previously tested in by Ursing et al. [50] and a standard dose (25 mg/kg, green shaded area) recommended for children [57]. Whilst these levels correspond to efficacious exposure for the treatment of malaria (i.e., erythrocytes in blood represent the target site), predicted concentrations of CQ in lung tissue do not achieve the anticipated minimum threshold for the so-called worst-case scenario. Even though a high probability of target attainment is predicted for total CQ lung tissue concentrations (Table 3), the evaluated regimens may not be efficacious even in the best-case scenario, if one considers free instead of total tissue concentrations (Figure 3).

**Figure 4:**
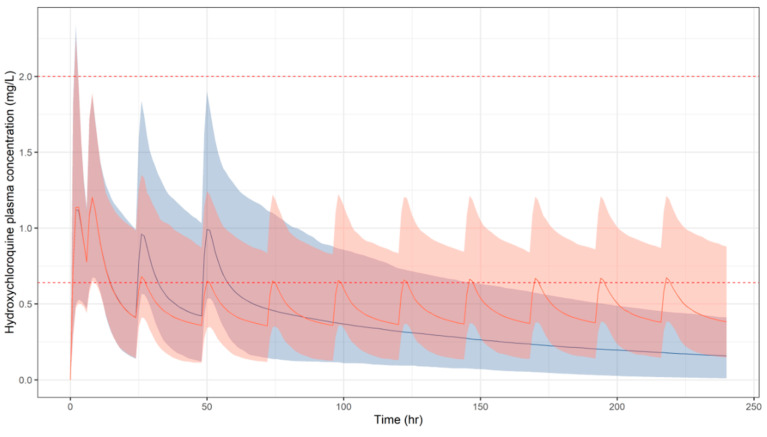
Simulated plasma HCQ concentration vs. time profiles following oral administration of the short (blue) and extended (orange) regimens used for the treatment of malaria. Solid lines and shaded areas depict the predicted median profile and corresponding 95% confidence intervals, respectively. Dotted red lines represent the safety thresholds of 0.64 and 2 mg/L, as reported in [53,54]. Results refer to a paediatric population (*n* = 963), with a body weight range between 12.5 and 100 kg. Dose and schedules are summarised in Table 2.

**Figure 5:**
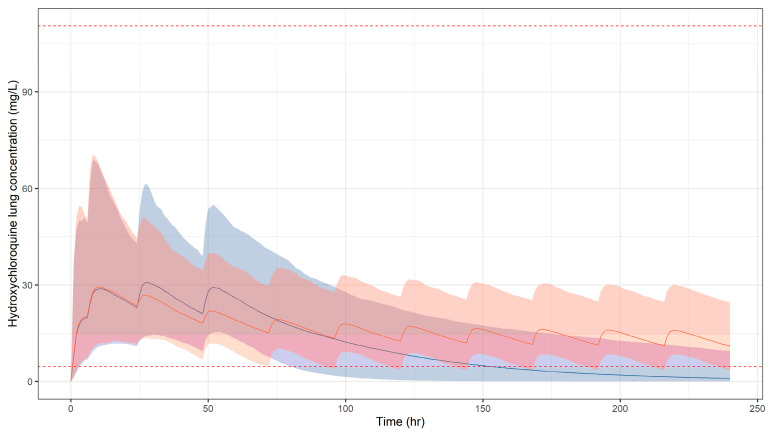
Simulated HCQ concentration vs. time profiles in lung tissue following oral administration of the short (blue) and extended (orange) regimens used for the treatment of malaria. Solid lines and shaded areas depict the predicted median profile and corresponding 95% confidence intervals, respectively. Dotted red lines represent the lowest (4.59 mg/L) and highest (110.5 mg/L) estimates for IC_95_. Results refer to a paediatric population (*n* = 963), with a body weight range between 12.5 and 100 kg. Dose and schedule details are summarised in Table 2.

In Figure 4, simulated HCQ blood concentration vs. time profiles are depicted over a treatment period of 10 days. Similarly to CQ, a high probability of target attainment is predicted in the best-case scenario only for total tissue concentrations following the extended dosing regimen (Figure 5, Table 3). It should be highlighted that whilst exposures in children are predicted to be well tolerated for currently recommended regimens of CQ, HCQ, concentrations in plasma are predicted to remain above the threshold associated with adverse events.

**Figure 6:**
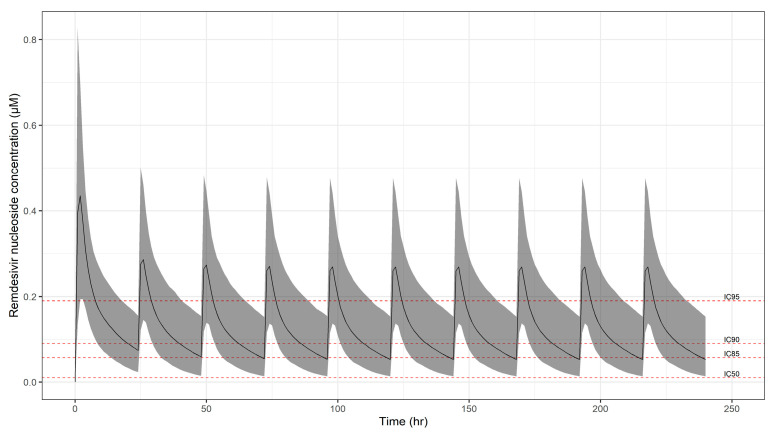
Simulated GS-441524 plasma concentration vs. time profiles following oral administration of RDV over a period of 10 days. Solid line and shaded area depict the predicted median profile and corresponding 95% confidence intervals, respectively. Dotted lines represent the extrapolated estimates for IC_50_ (0.006 mg/L), IC_85_ (0.034 mg/L), IC_90_ (0.054 mg/L), and IC_95_ (0.114 mg/L). Results refer to a paediatric population (*n* = 963), with a body weight range between 12.5 and 100 kg. Dose and schedule details are summarised in Table 2.

In Figure 6, the simulated concentration vs. time profiles of GS-441524 are depicted over a treatment period of 10 days. Even though a high probability of target attainment may be achieved at peak concentrations, systemic exposure to GS-441524 decreases rapidly during the dosing interval, with predicted low plasma and lung tissue levels even in the best-case scenario (Table 3).

## 4. Discussion

Unfortunately, this pandemic has shown how unprepared the clinical community is to embrace a paradigm which enforces quantitative principles as a requirement for repurposing compounds [11]. The large number of trials which have been terminated early due to lack of efficacy, futility, or safety concerns highlights the importance of shifting away from empirical dose selection [58]. In the current investigation, we sought to illustrate how population pharmacokinetic modelling and extrapolation are applied in conjunction with CTS to describe the systemic and putative lung tissue exposure in a paediatric population receiving CQ, HCQ, and RDV for the treatment of SARs-CoV-2 infection [59].

We have demonstrated not only how different data sources can be utilised, but also how uncertainty in PKPD parameter estimates (and ultimately the benefit–risk balance of an intervention) can be evaluated in a systematic manner before exposing vulnerable patients to a potentially harmful or non-efficacious treatment [60,61]. Our work also shows that available information on the pharmacokinetics, pharmacodynamics and safety could have been used to inform clinical trial design. The possibility of performing a preliminary evaluation of the predictive performance of the models by comparing the predicted exposure with observed data in paediatric patients across a similar age group [48,49,50] has contributed to further confidence in parameter precision. Of note were the predicted median (95%-CI) trough concentrations of HCQ (726.0 (263.4–1339) ng/mL), which showed complete overlapped with mean (SD) reported data (mean 665 (433) ng/mL) [49].

It is worth mentioning that the work presented here was implemented at the start of the pandemic in 2020, in parallel to efforts to identify suitable treatment options for adult patients with COVID-19. It became evident that despite the limited data and assumptions required to extrapolate in vitro findings to humans, modelling of the antiviral activity taking into account tissue equilibration kinetics offers a robust basis for the dose rationale in the target paediatric population. Such an in silico protocol represents a critical step for the evaluation of safety and efficacy of compounds before progressing into a real clinical trial. Regardless of the uncertainty about the correlation between viral inhibition in vitro and clinically meaningful improvement (i.e., survival, recovery after intubation), characterisation of drug disposition and prediction of systemic and target tissue exposure prevents clinical testing of doses or dosing regimens that have low probability of pharmacological success and/or are likely to be toxic [62]. In fact, integration of in vivo pharmacodynamic endpoints was beyond the scope of our framework, as it was designed to assess the probability of pharmacological success based on the available pre-clinical data at the time the first clinical protocols were being proposed. Importantly, in times of critical need, assessing in a strictly quantitative manner the probability of target attainment associated with a dose or dosing regimen should be a priority. In the event of an outbreak of a novel viral pandemic, it is likely that no in vivo human data will be available; we should prevent the empirical choices made by alarmed investigators at the start of the pandemic in 2020.

From a safety perspective, even with the safe use of CQ in malaria and HCQ in juvenile idiopathic arthritis [63], we found that higher doses are required to achieve and maintain drug levels at or above the thresholds for antiviral activity reported in vitro. Furthermore, even if there is no established threshold concentration for toxicity in children, it could have been anticipated that adverse events due to off-target systemic effects are likely to occur at higher dose levels, in particular ventricular tachyarrhythmias and QT interval prolongation [53,64]. Consequently, CQ and HCQ are unlikely to result in clinically relevant inhibition of viral replication at doses currently used for approved indications. Nevertheless, data extrapolation and model predictions seem to be aligned with the results reported by some authors (e.g., Aljayyoussi et al. [65]), but show discrepancies with others (e.g., Maharaj et al. [66]. Irrespective of the disagreement in modelling results or the effect of differences in experimental protocols on drug potency estimates, our analysis was meant to illustrate the pathway for informed decision-making, enhancing the integration of available data and prior knowledge. Given the contrasting results between the best- and worst-case scenarios, and the risks associated with higher doses of CQ or HCQ, neither compound should be tested against SARS-CoV-2 in children. This recommendation is supported by the fact that CQ and HCQ did lead to a dose-dependent increase in the QTc interval in SARS-CoV-2 adult patients [58,67,68,69].

Interestingly, recent findings from experimental models and randomised clinical trials seem to corroborate the aforementioned recommendations, i.e., that CQ and HCQ do not improve clinical outcomes in COVID-19 patients at currently approved doses [28,70]. Furthermore, our results show that RDV may show some antiviral activity at doses previously tested for Ebola virus infection, but it fails to reach the desirable target concentration for sustained, clinically relevant viral suppression. Higher doses than 5 mg/kg were not tested for RDV because of the lack of safety data available at the time of this investigation. Our initial results showed that the extrapolation of pharmacokinetics from rhesus macaques to humans underpredicted systemic exposures in adults by more than 50% [24]. On the other hand, this discrepancy could be explained by poor precision of the estimates used to extrapolate the disposition parameters to humans, an issue which was partly addressed by the sensitivity analysis. In fact, underexposure is one explanation as to why RDV has only been reported to improve the time to recovery [71,72].

## 5. Limitations and Clinical Implications

Viruses are intracellular pathogens which react to the antiviral activity of a drug if pharmacologically relevant concentrations are reached and maintained within the intracellular compartment. Our benchmark for anti-viral activity was based on the extrapolation of estimates of the extracellular concentrations from in vitro experiments [73], which lead to three important limitations. The first is that it remains unclear what the relationship is between extracellular and intracellular drug concentrations in lung tissue of subjects affected by SARS-CoV-2. Irrespective of the availability of physiologically-based pharmacokinetic models [74], this relationship cannot be validated without sampling drug concentrations in human lung tissue. Therefore, to overcome this limitation, we have used the lung tissue-to-blood partition coefficient as basis for the prediction of lung exposure. We acknowledge that this approach may not be accurate for drugs that are actively transported or heavily metabolised, since affinity for different pathways may vary significantly across species. Hence, our results for RDV should be interpreted with caution. The second limitation is that we did not perform a full sensitivity analysis on the inhibitory concentrations to explore the implications of varying thresholds and/or viral resistance. The working hypothesis assumed that targeting concentrations corresponding to approximately IC_95_ were required to translate viral inhibition into clinical improvement—i.e., to prevent severe complications like pneumonitis, multi-organ dysfunction, and coagulopathies. This threshold was selected based on previous examples in other viral diseases (HIV, hepatitis), which suggest exposure levels associated with maximum viral suppression for efficacy [75,76]. For the sake of clarity, we have investigated different doses and dosing regimens using single point estimates (e.g., IC_95_) and assuming a time-independent effect, with negligible mutation rate during the course of treatment [77]. In addition, CQ and HCQ in vitro potency estimates (IC_50_) varied by one order of magnitude depending on experimental conditions, causing uncertainty about the anticipated antiviral activity in vivo. This is important, since CQ and HCQ are drugs with a narrow therapeutic index. A third limitation is that there may also be differences in tissue equilibration kinetics in vivo. Moreover, we have excluded children younger than 2 years old to prevent the need for a more complex pharmacokinetic model including the effect of maturation and ontogeny, which are known to alter drug disposition in this age group [59]. Another point to consider is that our simulations were based on the demographics of a healthy paediatric population. However, pathophysiological changes induced by SARS-CoV-2 and/or co-morbidities and co-medications may alter pharmacokinetics and potentially treatment response in children. For example, a pro-thrombotic state has been observed in adults with SARs-CoV-2 infection, which may lower the ability for drugs to distribute to areas that are poorly perfused. It should further be acknowledged that if paediatric patients require cardiorespiratory support through extracorporeal membrane oxygenation (ECMO), the pharmacokinetic properties of many drugs may be significantly altered, necessitating dose adjustments [78]. Lastly, it should be highlighted that we have not investigated whether there is an optimal time frame for initiating treatment once infected to SARS-CoV-2, as seen with other viral infections [79,80,81]. Outcomes are expected to be worse for patients with higher disease progression, or higher viral load. In fact, no benefit has been observed when RDV is administered after the peak of viral infection in pre-clinical models of infection [82,83,84]. In adults, RDV has shown more pronounced benefits when given earlier in the infection [71], but it did not reverse the status of infection at day 12 when initiated 7 days after the onset of symptoms [85].

## 6. Conclusions

Regardless of the frailty of older adults affected by COVID-19, higher risk of death and/or long-term sequalae [86], infected children can also show vulnerability and are potentially exposed to complications. This investigation outlined the main steps for the implementation of a model-based approach for the repurposing of marketed products and investigational drugs for the treatment of SARS-CoV-2 infection, with especial focus on the rationale for the paediatric dose selection. As such, the concepts presented here illustrate how modelling, simulation, and extrapolation can be used to support paediatric drug discovery and development. Most importantly, this work provides the basis for the dose rationale in a paediatric investigation plan, which is required for regulatory approval of a new product or indication in adults in the European Union.

Whilst assumptions represent an essential step in translational research, the use of a model-based framework for the repurposing of compounds with potential antiviral activity against SARS-CoV-2 virus highlights the flaws of empirical testing of dose permutations in clinical trials. In summary, our work shows that paediatric patients from 2 to <18 years of age should not be treated CQ or HCQ for the treatment of COVID-19 due to the low probability of pharmacological success and obvious safety concerns. It also reveals that the use of approved doses of RDV for Ebola (and COVID-19 in adult patients) is unlikely to achieve the required clinically relevant concentrations to ensure inhibition of viral replication.

## Figures and Tables

**Table 1:** Mechanism of action, potency (IC_50_) and PKPD parameters (IC_85_, IC_90_, IC_95_) used as reference threshold or target level assumed to be required to achieving clinically relevant antiviral activity in vivo against the SARS-CoV-2 virus.

Drug	Mechanisms Associated with Antiviral Activity	Derived PKPD Parameters (mg/L)	Reported IC_50_ (Value or Range) (mg/L)	Reference
Chloroquine	CQ has been considered as a broad-spectrum antiviral drug. Although the exact mechanisms are not fully understood, CQ shows antiviral and immunomodulatory properties by altering the acidic conditions necessary for virus-to-cell fusion and entry into host cells. CQ is thought to disrupt this mechanism through the de-acidification of endosomal/lysosomal organelles, which orchestrate enzyme cleavage of the SARS-CoV-2 spike glycoprotein. Post entry, CQ is also reported to de-acidify the Golgi apparatus, which can affect post-translational processes, such as proteolysis and glycosylation, preventing escape of the virus from the host cell. Additionally, binding of the S-protein through angiotensin-converting enzyme 2 is downregulated by CQ.	IC_95_ = 6.87–145.25	0.36–7.64	[12,14]
Hydroxy-chloroquine	HCQ, a derivative of CQ, is a safer compound than CQ in animals, and is considered to exert the same mechanisms of anti-viral activity as CQ upon SARS-CoV-2.	IC_95_ = 4.59–110.5	0.242–5.81	[12,14]
Remdesivir	RDV’s antiviral activity, sterically interacting with the viral RNA-dependent RNA polymerase (RdRp) to induce delayed chain termination, has been shown in vitro against multiple coronaviruses. RDV also alters pan-CoV RdRp function by inhibiting viral replication even in settings with intact exonuclease proof-reading activity. Biochemical data from recombinant respiratory syncytial virus RdRp suggested the primary mechanism of action was through delayed chain termination.	IC_85_ = 0.034 IC_90_ = 0.054 IC_95_ = 0.114	0.006	[16]

**Table 2:** Currently approved doses and dosing regimens of chloroquine, hydroxychloroquine, and remdesivir included in the simulation scenarios.

Drug	Classification	Dosing Regimen
CQ *	Standard dose	Loading: 10 mg/kg on day 1 ^†^, 5 mg/kg 8 h later 5 mg/kg on days 2, 3 Total cumulative = 25 mg/kg over 3 days
	High dose	Loading: 10 mg/kg b.i.d. on days 1 ^†^ and 2 5 mg/kg b.i.d. on day 3 Total cumulative = 50 mg/kg over 3 days
HCQ *	Short course	10 mg/kg + 5 mg/kg after 6, 24 and 48 h
	Extended course	Loading: 10 mg/kg + 5 mg/kg after 6 h on day 1 ^†^ and 2.5 mg/kg at 24 h (day 2) 2.5 mg/kg q.d. thereafter for 8 days
RDV	FDA approved doses	5 mg/kg q.d. (infused over 30 min) on day 1 ^†^ and 2.5 mg/kg q.d. (infused over 30 min) for 9 subsequent days or 200 mg q.d. on day 1 ^†^ and 100 mg q.d. for 9 subsequent days **

* CQ and HCQ doses are expressed as free base; ^†^ Day 1 is the first treatment day, with the first dose being administered at time 0. ** RDV regimens varied according to body weight: 5 mg/kg (day 1) + 2.5 mg/kg (day 2 onwards) for patients with body weight < 40 kg, and 200 mg (day 1) + 100 mg (day 2 onwards) for patients with body weight ≥ 40 kg.

**Table 3:** Probability of target attainment for each drug and dosing regimen.

Drug	Regimen	Probability of Target Attainment (%)						
		Best Case Target Concentration *				Worst Case Target Concentration ^a^		
		Day 1	Day 3	Day 7	Day 10	Day 1	Day 3	Day 7
CQ	Total 25 mg/kg	100	100	100	-	0	0	0
	Total 50 mg/kg	100	100	100	-	0	0	0
HCQ	Short dose	100	99	41	-	0	0	0
	Extended dose	100	97	96	-	0	0	0
RDV	5 mg/kg + 2.5 mg/kg ^b^ or 200 mg + 100 mg	-	-	-	8 **	N/A		

* PTA calculated using IC_95_ estimates from experiments with the highest in vitro potency; ** PTA calculated using lower thresholds, i.e., IC_85_ and IC_90_ corresponds to 98% and 78%, respectively; ^a^ PTA calculated using IC_95_ estimates from experiments with the lowest in vitro potency; ^b^ RDV regimens varied according to body weight: 5 mg/kg (day 1) + 2.5 mg/kg (day 2 onwards) for patients with body weight < 40 kg, and 200 mg (day 1) + 100 mg (day 2 onwards) for patients with body weight ≥ 40 kg; N/A—not evaluated or not applicable to the specific drug or scenario.

## Supplementary Materials

The following are available online at https://www.mdpi.com/article/10.3390/pharmaceutics13081299/s1, Figure S1: Compartmental pharmacokinetic model of chloroquine. Table S1: Population pharmacokinetic model parameter estimates for chloroquine. Figure S2. Compartmental pharmacokinetic model of hydroxychloroquine in whole blood. Table S2. Population pharmacokinetic model parameter estimates for hydroxychloroquine in whole blood. Figure S3. Compartmental pharmacokinetic model of hydroxychloroquine in plasma. Table S3. Population pharmacokinetic model parameter estimates for hydroxychloroquine in plasma. Figure S4. Compartmental pharmacokinetic model of remdesivir. Table S4. Population pharmacokinetic model parameter estimates for remdesivir in plasma. Table S5. Chloroquine: Model-predicted vs. observed concentrations in paediatric patients. Table S6. Hydroxychloroquine. Model-predicted concentrations in paediatric patients. Table S7. Remdesivir metabolite (GS441524). Model-predicted vs. observed secondary PK parameters in adults. Figure S5. Comparison between model predictions and previously reported remdesivir concentrations in adults. Figure S6. Sensitivity analysis: model predictions in adults for different case scenarios. Table S8. Remdesivir metabolite (GS-441524). Model-predicted secondary PK parameters in children. Table S9. Remdesivir metabolite (GS-441524). Sensitivity analysis: model-predicted secondary PK parameters in children for different case scenarios. Table S10. Remdesivir metabolite (GS441524). Sensitivity analysis: probability of target attainment for different case scenarios. Table S11. NONMEM control stream file for the simulation of chloroquine concentrations in blood. Table S12. NONMEM control stream file for the simulation of hydroxychloroquine concentrations in blood. Table S13. NONMEM control stream file for the simulation of hydroxychloroquine concentrations in plasma. Table S14. NONMEM control stream file for the simulation of remdesivir concentrations in plasma.
