# Supplementary material for: Model-Informed Repurposing of Medicines for SARS-CoV-2: Extrapolation of Antiviral Activity and Dose Rationale for Paediatric Patients
Source: Pharmaceutics. 2021 Aug 19;13(8):1299. doi: 10.3390/pharmaceutics13081299 (PMC8399437; doi:10.3390/pharmaceutics13081299)
Supplement: Supplementary file 1 [file pharmaceutics-13-01299-s001.zip › pharmaceutics-1273201-SI.pdf]

pharmaceutics

MDPI

Supplementary Materials: Model-Informed Repurposing of Medicines for SARS-CoV-2: Extrapolation of Antiviral Activity and Dose Rationale for Paediatric Patients

Federico Romano, Salvatore D'Agate, and Oscar Della Pasqua

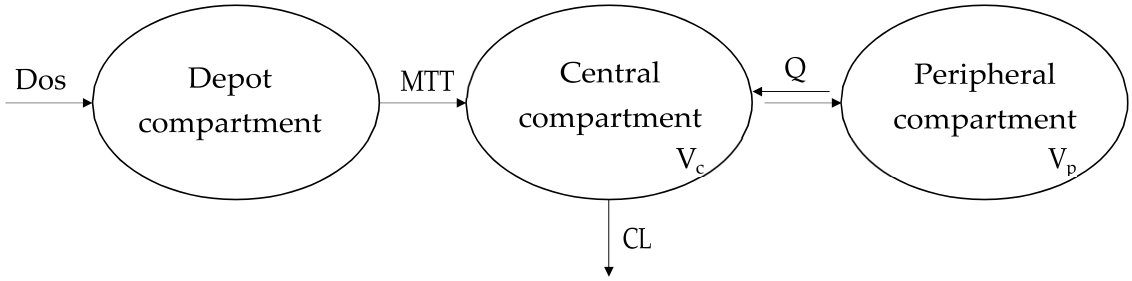

Figure S1. Compartmental pharmacokinetic model of chloroquine.

Table S1. Population pharmacokinetic model parameter estimates for chloroquine (CQ).

**Parameter**	**Estimate**
MTT (h)	0.773
CL/F (L/h)	6.13
V_C/F (L)	468
V_P/F (L)	1600
Q/F (L/h)	37.7
$t_{1/2}$ (days)	10.7
ω^2_{VP}	20.0
ω^2_F	19.4
σ^2_{prop}	0.401

Table adapted from Höglund et al. [1]. MTT mean transit time of the absorption, CL/F apparent clearance of CQ, V_C/F apparent volume of distribution for CQ central compartment, V_P/F apparent volume of distribution for CQ peripheral compartment, Q/F apparent intercompartmental clearance for CQ, $t_{1/2}$ half-life of CQ. ω, inter-individual variance; σ^2, intra-individual variance.

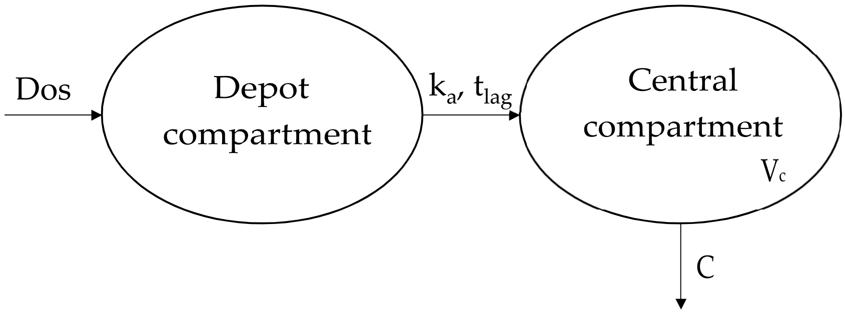

Figure S2. Compartmental pharmacokinetic model of hydroxychloroquine in whole blood.

Table S2. Population pharmacokinetic model parameter estimates for hydroxychloroquine in whole blood.

Parameter	Estimate
CL (L/h)	9.89
V (L)	605
k_a (h^{-1})	0.765
t_{lag} (h)	0.445
F	0.746
k_p	29
ω^2_{CL}	0.127
ω^2_V	0.25
ω^2_{ka}	0.94
σ^2_{prop}	0.044
σ^2_{add}	365

Table adapted from Carmichael et al. [2] CL, clearance; V, volume of distribution; k_a, absorption rate constant; t_{lag}, absorption lag constant; F, bioavailability; k_p, blood:lung ratio found in Chhonker et al. [3]; ω^2, inter-individual variance; σ^2, intra-individual variance. This model was used to extrapolate lung concentration using the blood:lung ratio parameter and to assess drug efficacy.

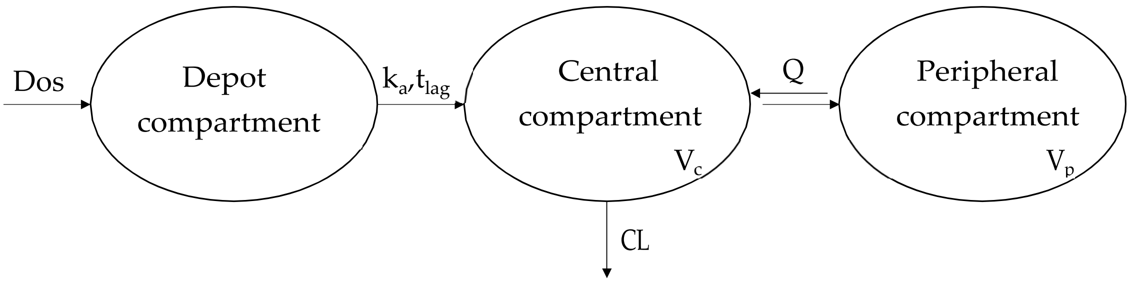

Figure S3. Compartmental pharmacokinetic model of hydroxychloroquine in plasma.

Table S3. Population pharmacokinetic model parameter estimates for hydroxychloroquine in plasma.

Parameter	Estimate
CL (L/h)	10.9
Vc (L)	437
k_a (h^{-1})	1.15
t_{lag} (h)	0.389
Q (L/h)	45.1
V_p (L)	1390
ω^2_{CL}	0.161
$\omega^2_{V_c}$	0.232
$\omega^2_{V_p}$	0.715
$\omega^2_{t_{lag}}$	0.0359
σ^2_{prop}	0.0729
σ^2_{add}	7.7

Table adapted from Lim et al. [4] CL, clearance; V_c, volume of distribution of the central compartment; k_a, absorption rate constant; t_{lag}, absorption lag time ; Q, intercompartmental clearance; V_p, volume of distribution of the peripheral compartment; ω^2, interindividual variance; σ^2, intra-individual variance. This model was used to assess safety threshold for systemic exposure.

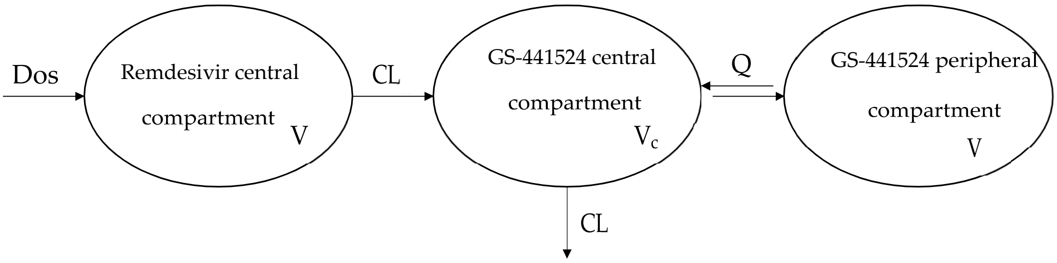

Figure S4. Compartmental pharmacokinetic model of remdesivir.

Table S4. Population pharmacokinetic model parameter estimates for remdesivir (RDV) in plasma.

Parameter	Estimate
CL RDV (L/h)	68.5
CL GS-441524 (L/h)	13.6
V RDV (L)	32.1
V_C GS-441524 (L)	74.3
Q GS-441524 (L/h)	22.7
V_P GS-441524 (L)	78.6
Proportional RUV (%)	13.7

Pharmacokinetic parameters: CL, clearance; V_C, volume of distribution of the central compartment; Q, intercompartmental clearance; V_P, volume of distribution of the peripheral compartment; ω^2, interindividual variance; σ^2, intra-individual variance.

To assess the predictive performance of the pharmacokinetic model, including modelling and extrapolation assumptions for chloroquine and hydroxychloroquine, blood concentrations at day 7 were compared with data reported by Ursing et al. [5]. Median area under the concentration vs. time curve over the treatment period ($AUC_{0–168}$), maximum concentration (C_{max}), steady state concentration (C_{ss}) and trough concentration (C_{min} or C_{tau}) in plasma and lung were derived when possible. Plasma concentrations were derived by taking into account the blood-to-plasma ratio [6].

Table S5. Chloroquine: Model-predicted vs observed concentrations in paediatric patients.

		Model predictions *				Reported data
**Drug**	**Regimen**	**Median lung $AUC_{0–168}$ (mgh/L)**	**Plasma C_{max} (mg/L)**	**Median plasma $AUC_{0–168}$ (mgh/L)**	**Median blood concentrations on day 7 (mg/L)**	**Blood concentrations (mg/L) on day 7 [a,b]**
CQ	Total 25mg/kg	4762 (3253–6888)	0.23 (0.2–0.36)	19.2 (12.8–27.2)	0.35 (0.25–0.53)	0.1–0.32
	Total 50mg/kg	9397 (6511–13322)	0.42 (0.28–0.63)	37.6 (26.4–53 6)	0.77 (0.53–1.1)	0.19–0.79

[a] IQR–interquartile range; [b] Clinical data set reference for 2–14 year old children, based on a maximum weight of 29 kg–Ursing et al. [5]; * Values computed over 963 virtual paediatric subjects, ranging from 2–18 years postnatal age; Values between parentheses represent the 95% confidence intervals.

Table S6. Hydroxychloroquine: Model-predicted concentrations in paediatric patients.

		Model-predictions *					
**Drug**	**Regimen**	**Median lung concentration (mg/L)**			**Median lung $AUC_{0–168}$ (mgh/L)**	**Median plasma C_{max} (mg/L) on Day 1**	**Median plasma $AUC_{0–168}$ (mgh/L)**
		**Day 1**	**Day 3**	**Day 7**			
HCQ	Short dose	22.9 (11.0–42.9)	20.2 (7.6–41.1)	3.5 (0.0–15.5)	2757 (1418–5360)	1.2 (0.7–2.3)	80 (39–157)
	Extended dose	23.5 (11.6–44.4)	15.1 (4.5–29.7)	11.5 (3.6–25.3)	3079 (1685–5538)	1.2 (0.7–2.3)	83 (46–164)

* Values computed over 963 virtual paediatric subjects, ranging from 2–18 years postnatal age; Values between parentheses represent the 95% confidence intervals.

For remdesivir, model predictions were compared to the values reported in [7]. This evaluation is based on a regimen including a 200 mg IV dose (infused over 30 min) on Day 1 and 100 mg for 9 subsequent days once daily (infused over 30 min). AUC was derived after the first 200 mg dose. C_{max} and C_{tau} were calculated at steady state after a 100 mg dose.

Table S7. Remdesivir metabolite (GS441524): Model-predicted vs observed secondary PK parameters in adults [3].

Drug	Regimen	Parameter	Predicted mean * (ng/mL)	Reported mean (ng/mL) [CV%]
GS-441524	200 mg	$AUC_{0–24}$	842 (443–1398)	2190 [19]
	100 mg	$C_{max,ss}$	56 (27–101)	145 [19]
		$C_{tau,ss}$	15 (4–35)	69 [18]

* Values computed over 963 virtual paediatric subjects, ranging from 2–18 years postnatal age. Values between parentheses represent the 95%-confidence.

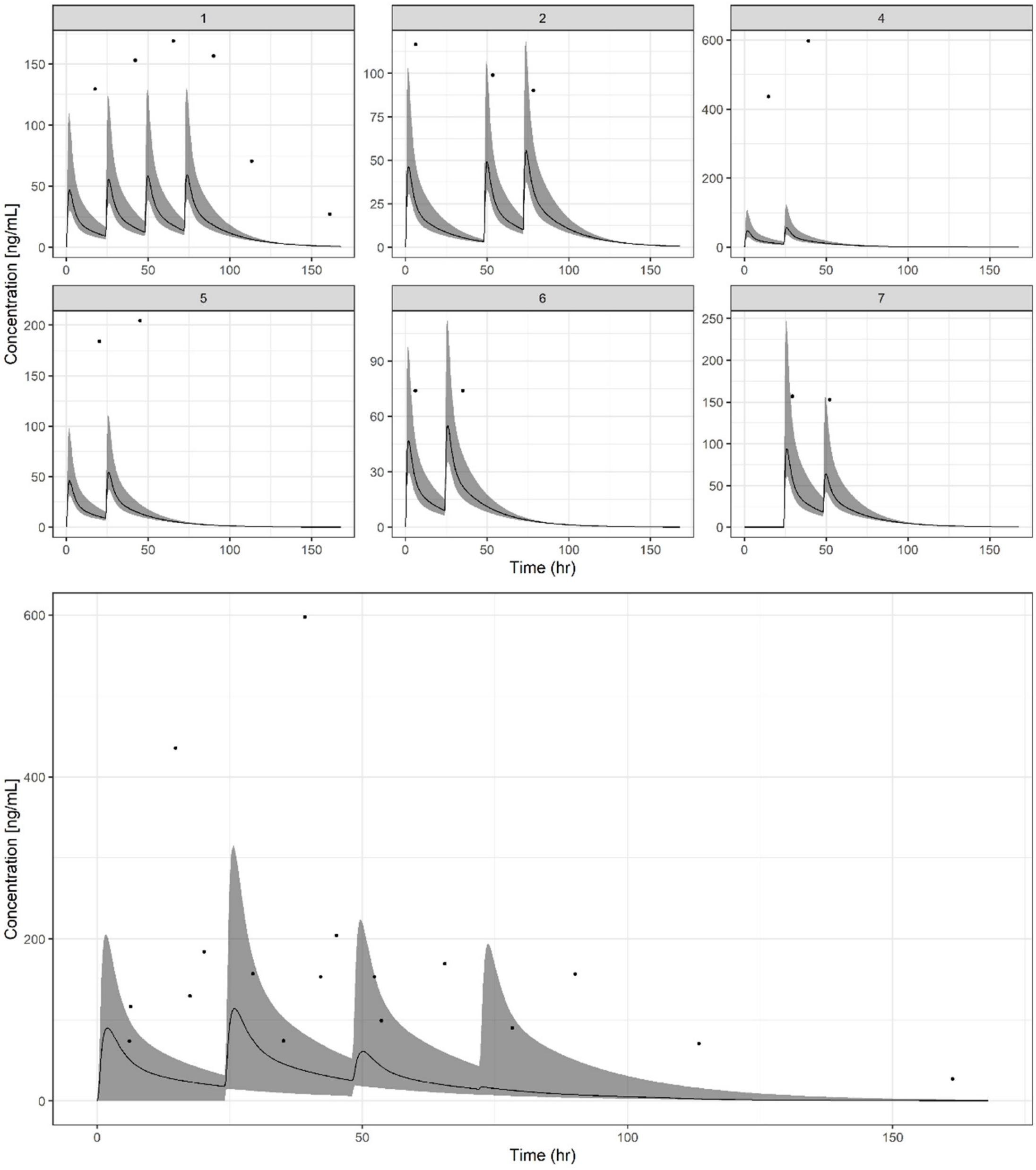

Figure S5. Comparison between model predictions and previously reported remdesivir concentrations in adults.

Visual predictive checks show the comparison between model predicted median concentrations (black line) and the corresponding 95% confidence intervals (grey area). Observed data are depicted by the black dots [8]. Model predictions were based on 1000 simulations. Subjects in the data set were assigned a body weight randomly sampled from a normal distribution with a mean of 70 kg and standard deviation of 20 kg.

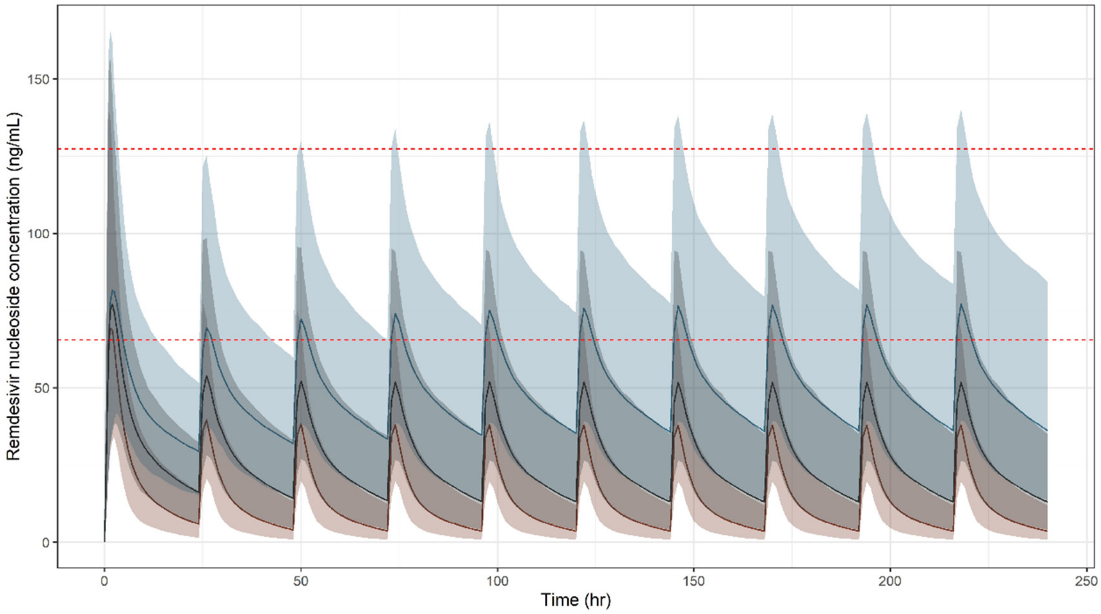

Figure S6. Sensitivity analysis: model predictions in adults for different case scenarios.

GS-441524 concentration vs. time profile in adults receiving 200 mg RDV on day 1 followed by 100 mg daily for 9 days. Grey line and shaded area represent the median and 95% confidence intervals (CI) obtained using the model estimates extrapolated from rhesus macaques. Blue and brown lines show the median obtained using, respectively, model estimates with elimination rate constant 2-fold smaller and larger than the value extrapolated by allometric scaling of preclinical data. Red dotted lines represent the 95% CI of previously reported state concentrations (C_{ss}) in adults [7]. Shaded area depicts the 95% CI.

Table S8. Remdesivir metabolite (GS-441524): Model-predicted secondary PK parameters in children.

Drug	Model predictions *		Reported data
	Median plasma AUC_{0-24} (μM×h)	Median plasma C_{max} (μM)	Median plasma C_{min} (μM)
GS-441524	2.891 (1.404–5.672)	0.280 (0.141–0.484)	0.053 (0.014–0.153)

* Values computed over 963 virtual paediatric subjects, ranging from 2–18 years postnatal age. AUC, C_{max} and C_{min} were calculated at steady state. Values between parentheses represent the 95% confidence intervals.

Table S9. Remdesivir metabolite (GS-441524). Sensitivity analysis: model-predicted secondary PK parameters in children for different case scenarios.

Scenario	Model predictions *		Reported data
	Median plasma AUC_{0-24} (μM×h)	Median plasma C_{max} (μM)	Median plasma C_{min} (μM)
Elimination rate constant (2-fold larger)	1.445 (0.702–2.837)	0.225 (0.106–0.400)	0.013 (0.002–0.050)
Elimination rate constant (2-fold smaller)	5.781 (2.808–11.202)	0.407 (0.209–0.694)	0.158 (0.056–0.376)

*Values computed over 963 virtual paediatric subjects, ranging from 2–18 years postnatal age. AUC, C_{max} and C_{min} were calculated at steady state. Values between parentheses represent the 95% confidence intervals. Variations in metabolite formation rate did not produce any clinically significant difference.

Table S10. Remdesivir metabolite (GS441524). Sensitivity analysis: probability of target attainment for different case scenarios.

Scenario *	Probability of target attainment (%)			
	IC_{50}	IC_{85}	IC_{90}	IC_{95}
Elimination rate constant (2-fold larger)	100	56	12	0
Elimination rate constant (2-fold smaller)	100	100	99	74

* Variations in metabolite formation rate did not produce any clinically significant difference.

Table S11. NONMEM control stream file for the simulation of chloroquine concentrations in blood.

```
$PROBLEM 1 ;. 1 comp CQ,1 comp DECQ – Control stream includes compartments describing CQ
metabolite (DEC), which was not included in the final analysis.
$INPUT ID TIME DV MDV EVID ARM WT CMT AMT II ADDL DOSE
$DATA Paediatric_dosing.csv IGNORE=#
$SUBROUTINE ADVAN5 TRANS1; TOL=6
$MODEL
COMP=(1) ; Absorption compartment
COMP=(2) ; Central compartment CQ
COMP=(3) ; Central compartment DEC
COMP=(4) ; Peripheral compartment DEC
COMP=(5) ; Peripheral compartment CQ
COMP=(6) ; Transit compartment
$PK
TVQ2 = THETA(1) * (WT/70)**0.75;
TVV2 = THETA(2) * (WT/70)**1
TVCL = THETA(3) * (WT/70)**0.75
TVV3 = THETA(4) * (WT/70)**1;
TVMT = THETA(5) ;
TVQ = THETA(6) * (WT/70)**0.75 ;
TVV4 = THETA(7) * (WT/70)**1;
TVV5 = THETA(8) * (WT/70)**1;
TVQ1 = THETA(11) * (WT/70)**0.75;
TVF1 = THETA(12);
Q2 = TVQ2* EXP(ETA(1)) ;
V2 = TVV2* EXP(ETA(2))
CL = TVCL* EXP(ETA(3)) ;
V3 = TVV3* EXP(ETA(4)) ;
MT = TVMT* EXP(ETA(5)) ;
Q = TVQ * EXP(ETA(6)) ;
V4 = TVV4* EXP(ETA(7)) ;
V5 = TVV5* EXP(ETA(8)) ;
CLM = 0.176*Q2
Q1 = TVQ1* EXP(ETA(9)) ;
F1 = TVF1* EXP(ETA(11)) ;
```

```
nn=1 ; calculating KTR based on the MTT estimates, nn=the number of transit compartments
KTR = (nn+1)/MT
K16 = KTR
K62 = KTR
K20 = Q2/V2
K30 = CL/V3
K24 = Q/V2
K42 = Q/V4
K23 = CLM/V2
K35 = Q1/V3
K53 = Q1/V5
S2=V2
S3=V3
$ERROR
IPRED = A(2)/V2
W = THETA(9)
;Metabolite error model
IF(CMT.EQ.3) THEN
IPRED = A(3)/V3
W = THETA(10)
ENDIF
IPRED=IPRED
IRES = DV - IPRED
IWRES = IRES/W
Y = IPRED + W*EPS(1)
$THETA
(0, 6.13) ; (1.CL/F L/h CQ (Q2))
(0, 469) ; (2 V2/F L)
(0, 2.05) ; (3.CLM/F L/h M (CL))
(0, 2.31) ; (4.V3/F L M)
(0, 0.945) ; (5.MT /h)
(0, 37.8) ; (6.Q /L/h (parent))
(0, 1600) ; (7.V4 L)
(0, 258) ; (8. V5)
(0, 0.396) ; (9.Add (prop) error)
(0, 0.428) ; (10.Add (prop) error)
(0, 1.46) ; (11. Q (metabolite))
(1) FIX ;
$OMEGA
0 FIX
0 FIX
0 FIX
0.129
```

```
0 FIX
0 FIX
0.0375
0.566
0 FIX
1 FIX
0.0381
$SIGMA 1 FIX
$SIM ONLYSIM (2345) NSUBPROBLEMS=1
$TABLE ID TIME CMT IPRED IRES IWRES CWRES MDV NOPRINT ONEHEADER FILE=sdtabPaed
```

Table S12. NONMEM control stream file for the simulation of hydroxychloroquine concentrations in blood.

```
$PROBLEM PK
$INPUT ID TIME DV MDV AGE SEX WT HT BMI HCT AMT II ADDL CMT
$DATA HCQ_dataset_children_standard_treatment_base.csv IGNORE=@
$SUBROUTINES ADVAN6 TOL=3
$ABBREVIATED COMRES=1
$MODEL COMP=(ABS, DEFDOSE) COMP=(CENTRAL, DEFOBS) COMP=(AUC)
$PK
CL = THETA(1)*(WT/70)**0.75*EXP(ETA(1))
V = THETA(2)*(WT/70)*EXP(ETA(2))
KA = THETA(3)*EXP(ETA(3))
ALAG1 = THETA(4)
F1 = THETA(5)
S2 = V/1000
IF (NEWIND.LE.1) COM(1)=-1
$DES
DADT(1) = -KA*A(1)
DADT(2) = KA*A(1) - CL/V*A(2)
DADT(3) = A(2)
$ERROR
CP = A(2)/S2
IF (CP.GT.COM(1)) COM(1)=CP
CMAX = COM(1)
AUC = A(3)/S2
IPRED = F
W = SQRT(THETA(6)**2*IPRED**2 + THETA(7)**2)
Y = IPRED + W*EPS(1)
IRES = DV-IPRED
IWRES = IRES/W
$THETA
(0, 9.89) ; CL
(0, 605) ; V
(0, 0.765) ; KA
```

```
(0, 0.445) ; ALAG1
(0, 0.746) ; F1
(0, 0.209) ; Prop.RE (sd)
(0, 19.104) ; Add.RE (sd)
$OMEGA
(0.127) ; IIV CL
(0.25) ; IIV V
(0.94) ; IIV KA
$SIGMA
1 FIX ; Proportional error PK
$SIM (12345) (54321) ONLYSIM NSUB=1
$TABLE ID TIME DV MDV IPRED CWRES AGE SEX WT HT BMI HCT V CL CMAX CMT AUC ONE-
HEADER
NOPRINT FILE=sdtab0032
```

Table S13. NONMEM control stream file for the simulation of hydroxychloroquine concentrations in plasma.

```
$PROBLEM PK
$INPUT ID TIME DV MDV AGE SEX WT HT BMI HCT AMT II ADDL CMT
$DATA HCQ_dataset_children_standard_treatment_base.csv IGNORE=@
$SUBROUTINES ADVAN6 TOL=3
$MODEL COMP=(ABS, DEFDOSE) COMP=(CENTRAL, DEFOBS) COMP=(PERIPH) COMP=(AUC)
$PK
CL = THETA(1)*(WT/70)**0.75*EXP(ETA(1))
V2 = THETA(2)*(WT/70)*EXP(ETA(2))
KA = THETA(3)
Q = THETA(4)*(WT/70)**0.75
V3 = THETA(5)*(WT/70)*EXP(ETA(3))
ALAG1 = THETA(6)*EXP(ETA(4))
S2 = V2/1000
S3 = V3/1000
K20 = CL/V2
K23 = Q/V2
K32 = Q/V3
$DES
DADT(1) = -KA*A(1)
DADT(2) = KA*A(1) - (K20 + K23)*A(2) + K32*A(3)
DADT(3) = K23*A(2) - K32*A(3)
DADT(4) = A(2)
$ERROR
AUC = A(4)/S2
IPRED = F
W = SQRT(THETA(7)**2*IPRED**2 + THETA(8)**2)
Y = IPRED + W*EPS(1)
IRES = DV-IPRED
```

```
IWRES = IRES/W
$THETA
(0, 10.9) ; CL
(0, 437) ; V2
(0, 1.15) ; KA
(0, 45.1) ; Q
(0, 1390) ; V3
(0, 0.389) ; ALAG1
(0, 0.27) ; Prop.RE (sd)
(0, 2.77) ; Add.RE (sd)
$OMEGA
(0.161) ; IIV CL
(0.232) ; IIV V2
(0.715) ; IIV V3
(0.0359) ; IIV ALAG1
$SIGMA
1 FIX ; Error PK
$SIM (12345) (54321) ONLYSIM NSUB=1
$TABLE ID TIME DV MDV IPRED CP CWRES AGE SEX WT HT BMI HCT CMT AUC ONEHEADER
NOPRINT
FILE=sdtab0033
```

Table S14. NONMEM control stream file for the simulation of remdesivir concentrations in plasma.

```
$PROBLEM PK
$INPUT ID TIME DV MDV CMT RATE AGE SEX WT HT BMI HCT AMT II ADDL
$DATA Remdesivir_children_sim_5mgkg_final_mM.csv IGNORE=@
$SUBROUTINES ADVAN6 TOL=3
$MODEL COMP=(PARENT) COMP=(NUC) COMP=(NUC2) COMP=(AUC)
$ABBREVIATED COMRES=1
$PK
CLM = THETA(1)*(WT/7.7)**0.75*EXP(ETA(1))
CL = THETA(2)
CL20 = THETA(3)*(WT/7.7)**0.75*EXP(ETA(2))
V1 = THETA(4)*(WT/7.7)*EXP(ETA(3))
V2 = THETA(5)*(WT/7.7)*EXP(ETA(4))
Q = THETA(6)*(WT/7.7)**0.75
V3 = THETA(7)*(WT/7.7)
KM = CLM/V1
KE = CL/V1
K20 = CL20/V2
K23 = Q/V2
K32 = Q/V3
S1 = V1
S2 = V2
```

```
D1 = 0.5
$DES
DADT(1) = - KM*A(1) - KE*A(1) ; Parent compartment [microM]
DADT(2) = KM*A(1) - K23*A(2) + K32*A(3) - K20*A(2) ; Metabolite compartment [microM]
DADT(3) = K23*A(2) - K32*A(3)
DADT(4) = A(2)
CMET = A(2)/S2
IF (CMET.GT.COM(1)) COM(1)=CMET
$ERROR
IF (CMT.EQ.1) IPRED = A(1)/S1
IF (CMT.EQ.2) IPRED = A(2)/S2
Y = IPRED*(1 + EPS(1))
CMAX = COM(1)
AUC = A(4)/S2
$THETA
(0, 68.5) ; CLM
(0) FIX ; CL
(0, 13.6) ; CL20
(0, 32.1) ; V1
(0, 74.3) ; V2
(0, 22.7) ; Q
(0, 78.6) ; V3
$OMEGA
0.1 FIX
0.1 FIX
0.1 FIX
0.1 FIX
$SIGMA
0.0187
$SIM (12345) (54321) ONLYSIM
$TABLE ID TIME DV CMT IPRED CWRES MDV CMAX AUC CLM CL20 Q V1 V2 V3 WT FILE=sdtab0033
NOPRINT ONEHEADER
```

References

1. Höglund, R.; Moussavi, Y.; Ruengweerayut, R.; Cheomung, A.; Äbelö, A.; Na-Bangchang, K. Population pharmacokinetics of a three-day chloroquine treatment in patients with Plasmodium vivax infection on the Thai-Myanmar border. *Malar. J.* **2016**, *15*, 1–9, doi:10.1186/s12936-016-1181-1.
2. Carmichael, S.J., B. Charles, and S.E. Tett, Population pharmacokinetics of hydroxychloroquine in patients with rheu-matoid arthritis. *Ther. Drug Monit.* **2003**, *25*, 671–681.
3. Chhonker, Y.S.; Sleightholm, R.L.; Li, J.; Oupický, D.; Murry, D.J. Simultaneous quantitation of hydroxychloroquine and its metabolites in mouse blood and tissues using LC–ESI–MS/MS: An application for pharmacokinetic studies. *J. Chromatogr. B* **2018**, *1072*, 320–327, doi:10.1016/j.jchromb.2017.11.026.
4. Lim, H.-S.; Im, J.-S.; Cho, J.-Y.; Bae, K.-S.; Klein, T.A.; Yeom, J.-S.; Kim, T.-S.; Choi, J.-S.; Jang, I.-J.; Park, J.-W. Pharmacokinetics of Hydroxychloroquine and Its Clinical Implications in Chemoprophylaxis against Malaria Caused by Plasmodium vivax. *Antimicrob. Agents Chemother.* **2009**, *53*, 1468–1475, doi:10.1128/aac.00339-08.
5. Ursing, J., et al., Chloroquine is grossly underdosed in young children with malaria: Implications for drug resistance. PLOS ONE, **2014**, *9*, e86801, doi:10.1371/journal.pone.0086801.
6. Smit, C., et al., Chloroquine for SARS-CoV-2: Implications of its unique pharmacokinetic and safety properties. *Clin. Pharmacokinet*. **2020**, *59*, 659–669, doi: 10.1007/s40262-020-00891-1.
7. Humeniuk, R.; Mathias, A.; Kirby, B.J.; Lutz, J.D.; Cao, H.; Osinusi, A.; Babusis, D.; Porter, D.; Wei, X.; Ling, J.; et al. Pharmacokinetic, Pharmacodynamic, and Drug-Interaction Profile of Remdesivir, a SARS-CoV-2 Replication Inhibitor. *Clin. Pharmacokinet.* **2021**, *60*, 569–583, doi:10.1007/s40262-021-00984-5.
8. Reckers, A.; Wu, A.H.B.; Ong, C.M.; Gandhi, M.; Metcalfe, J.; Gerona, R. A combined assay for quantifying remdesivir and its metabolite, along with dexamethasone, in serum. *J. Antimicrob. Chemother.* **2021**, *76*, 1865–1873, doi:10.1093/jac/dkab094.
